# Regulatory elements and transcriptional control of chicken *vasa* homologue (*CVH*) promoter in chicken primordial germ cells

**DOI:** 10.1186/s40104-016-0133-5

**Published:** 2017-01-13

**Authors:** So Dam Jin, Bo Ram Lee, Young Sun Hwang, Hong Jo Lee, Jong Seop Rim, Jae Yong Han

**Affiliations:** 1 Department of Agricultural Biotechnology, Research Institute of Agriculture and Life Sciences, College of Agriculture and Life Sciences, Seoul National University, Seoul, 08826 South Korea; 2 Institute for Biomedical Sciences, Shinshu University, Minamiminowa, Nagano 399-4598 Japan

**Keywords:** Chicken, Chicken *vasa* homologue, Primordial germ cell, Regulatory element, siRNA-mediated knockdown

## Abstract

**Background:**

Primordial germ cells (PGCs), the precursors of functional gametes, have distinct characteristics and exhibit several unique molecular mechanisms to maintain pluripotency and germness in comparison to somatic cells. They express germ cell-specific RNA binding proteins (RBPs) by modulating tissue-specific *cis*- and *trans*-regulatory elements. Studies on gene structures of chicken *vasa* homologue (*CVH*), a chicken RNA binding protein, involved in temporal and spatial regulation are thus important not only for understanding the molecular mechanisms that regulate germ cell fate, but also for practical applications of primordial germ cells. However, very limited studies are available on regulatory elements that control germ cell-specific expression in chicken. Therefore, we investigated the intricate regulatory mechanism(s) that governs transcriptional control of *CVH*.

**Results:**

We constructed green fluorescence protein (GFP) or luciferase reporter vectors containing the various 5′ flanking regions of *CVH* gene. From the 5′ deletion and fragmented assays in chicken PGCs, we have identified a *CVH* promoter that locates at −316 to +275 base pair fragment with the highest luciferase activity. Additionally, we confirmed for the first time that the 5′ untranslated region (UTR) containing intron 1 is required for promoter activity of the *CVH* gene in chicken PGCs. Furthermore, using a transcription factor binding prediction, transcriptome analysis and siRNA-mediated knockdown, we have identified that a set of transcription factors play a role in the PGC-specific *CVH* gene expression.

**Conclusions:**

These results demonstrate that *cis*-elements and transcription factors localizing in the 5′ flanking region including the 5′ UTR and an intron are important for transcriptional regulation of the *CVH* gene in chicken PGCs. Finally, this information will contribute to research studies in areas of reproductive biology, constructing of germ cell-specific synthetic promoter for tracing primordial germ cells as well as understanding the transcriptional regulation for maintaining germness in PGCs.

## Background

Primordial germ cells (PGCs) that emerge during early embryogenesis undergo a series of developmental events, such as specification, migration, and differentiation, to produce a new organism in the next generation [1, 2]. They express RNA binding proteins (RBPs) by modulating tissue-specific *cis*- and *trans*-regulatory elements and have specialized genetic programs distinct from those of other somatic cells for maintaining their unique characteristics [3–5]. Significant efforts have been made to elucidate the detailed molecular mechanisms regulating transcriptional control in germ cells [6–8]. At the transcriptional level, certain genes are effectively silenced, whereas other genes are exclusively expressed to maintain the levels of germline-expressed gene products [9, 10].

In chicken, PGCs separate from the epiblast in the blastoderm at Eyal-Giladi and Kochav stage X, which consist of 40,000 to 60,000 undifferentiated embryonic cells, and translocate into the hypoblast area of the pellucida [11, 12]. During gastrulation, they circulate through the vascular system and finally settle in the gonadal anlagen. After the arrival of PGCs, these cells continue to proliferate until they enter meiosis. This development of the PGC lineage is a highly complex process that is controlled by the coordinated action of many key factors, such as the expression and regulation of germline-specific genes [13, 14].

Evolutionarily conserved germ cell-specific *vasa* has been characterized in germ cells in several organisms, including chicken [15–17], zebrafish [18], mouse [19], and human [20]. Several studies have demonstrated that *vasa* plays critical roles in germ cell specification, supporting germ line development, translational control of transcribed genes, and RNA processes involving the biosynthesis of PIWI-interacting RNAs (piRNAs) in germ cells at the post-transcriptional level [21–26]. However, the intricate regulatory mechanism(s) that governs transcriptional control of *vasa* expression during chicken germline development has yet to be investigated in detail.

Understanding the cellular and molecular mechanisms that regulate germ cell-specific gene expression during PGC development is critical for the practical use of genetic modifications and germ-cell biology. In the current study, to characterize the promoter of chicken *vasa* homologue (*CVH*) for inducing germ cell-specific gene expression, we conducted 5′ deletion and fragment assays using both enhanced green fluorescent protein (eGFP) and NanoLuc luciferase expression vector. Furthermore, we investigated the predicted putative binding of transcription factors (TFs) on the promoter for *CVH*. Finally, we demonstrated that the transcriptional control of *CVH* expression through *cis*-elements and TFs is important for germ cell-specific gene expression in chicken PGCs.

## Methods

### Experimental designs, animals and animal care

This study was designed with the aim of investigating the *cis*- and *trans*-regulatory elements for modulating the transcription of *CVH* gene in chicken PGCs through dual luciferase assay and transcriptome analysis. The care and experimental use of chickens were approved by the Institute of Laboratory Animal Resources, Seoul National University (SNU-150827-1). The chickens were maintained in accordance with a standard management program at the University Animal Farm, Seoul National University, Korea. The procedures for animal management, reproduction, and embryo manipulation adhered to the standard operating protocols of our laboratory.

### Construction of eGFP and NanoLuc luciferase expression vectors controlled by *CVH* promoters of different sizes

For construction of eGFP expression vectors, the 5′ flanking regions of the *CVH* gene (NM_204708.2) were amplified using genomic DNA extracted from adult chicken blood, and subsequently inserted into the pGEM T easy vector (Promega, Madison, WI, USA). Primer sets were used to clone fragments of the *CVH* promoter of different sizes (Table 1). The eGFP coding sequence and polyadenylated (Poly-A) tail were inserted into the clone vectors including *CVH* promoter using the restriction enzymes *SpeI* and *NdeI*. For the construction of NanoLuc luciferase expression vectors, different lengths of the 5′ upstream region of the *CVH* gene were inserted between the *KpnI* and *XhoI* sites of pNL1.2 vectors (Promega).

**Table 1:** List of primer sequences used for cloning of the *CVH* promoter

Primer sets	Primer sequence (5′ → 3′)
CVH −1,575 bp_F	GACACAGCTTTCCCACGTGAG
CVH −1,231 bp_F	TGGCCACGTGCTATCATATTAGT
CVH −625 bp_F	CTCTGATCATGCCTGCAGCC
CVH −316 bp_F	CAGGACAGGCCTAGGGACAGA
CVH −227 bp_F	AGCATAAACAGGGAAAGCGC
CVH −135 bp_F	GCGCCACCTTCTCACCCC
CVH +25 bp_F	GCTATTTGGAGCGGAGAGTGAAA
CVH promoter_R	AGCGAATGCCAGCAGCC
CVH −135/+222_R	CGCCCTGACGCCACCAT
CVH −135/+162_R	AGCACGCACTGCCCTTGC
eGFP poly A _F	ACTAGTCCGCGGATGGTGAGCAAG
eGFP poly A_R	CATATGGACGTCTCCCCAGCATGCC

### Culture of chicken PGCs and DF-1

Chicken PGCs were cultured in accordance with our standard procedure [2]. Briefly, PGCs from White Leghorn embryonic gonads at 6 days old (Hamburger-Hamilton stage 28) were maintained in knockout Dulbecco’s Modified Eagle’s Medium (DMEM) (Gibco, Grand Island, NY, USA) supplemented with 20% fetal bovine serum (FBS) (Hyclone, South Logan, UT, USA), 2% chicken serum (Sigma-Aldrich, St. Louis, MO, USA), 1× nucleosides (Millipore, Billerica, MA, USA), 2 mmol/L L-glutamine (Gibco), 1× nonessential amino acids (Gibco), β-mercaptoethanol (Gibco), 1 mmol/L sodium pyruvate (Gibco), and 1× antibiotic-antimycotic (Gibco). Human basic fibroblast growth factor (bFGF) (Koma Biotech, Seoul, Korea) at 10 ng/mL was used for PGC self-renewal. The cultured PGCs were subcultured onto mitomycin-inactivated mouse embryonic fibroblasts at 5- to 6-day intervals by gentle pipetting without any enzyme treatment. For DF-1, the cells were maintained in DMEM with high glucose (Hyclone), 10% FBS, and 1× antibiotic-antimycotic. Cultured cells were grown at 37 °C in a 5% CO_2_ incubator.

### In vitro transfection

In vitro transfection was performed using Lipofectamine 2000 in accordance with the manufacturer’s instructions (Invitrogen, Carlsbad, CA, USA). For expression analysis of eGFP, the constructed *CVH* promoter vector (1 μg) and 2 μL of Lipofectamine 2000 were separately diluted with 50 μL of Opti-MEM I reduced serum medium (Invitrogen) and incubated at room temperature for 5 min. Liposome-DNA solutions were then mixed and incubated at room temperature for 20 min to form the lipid-DNA complex. Liposome-DNA complex solution was added to 2.5 × 10^5^ cultured PGCs in 500 μL of PGC culture medium. Transfected cells were incubated for 24 h without feeders. After incubation, cells were analyzed using a fluorescence microscope.

### Luciferase reporter assay

Nano-Glo Dual-Luciferase Reporter Assay System (Promega) was used to measure the *CVH* promoter activities. The prepared cells were seeded in a 96-well plate and co-transfected with pGL4.53 firefly luciferase (Fluc) and pNL1.2 (NlucP/CVH RE) NanoLuc luciferase (Nluc) plasmid using Lipofectamine 2000 (Invitrogen). The transfected cells were then lysed with lysis buffer with Fluc substrate and incubated on an orbital shaker for 3 min. Fluc signals were then quenched, followed by reaction with Nluc substrate. The signals in arbitrary unit (AU) from both Nluc and Fluc were measured using a luminometer (Glomax-Multi-Detection System; Promega). The promoter activities were calculated by the ratio of the respective AU values of Nluc/Fluc. pNL1.2, an empty vector, was used as a negative control.

### Prediction of putative transcriptional binding elements by in silico sequence analysis

The 591-base pair (bp) fragment (−316/+275) of the *CVH* promoter that had the highest activity was analyzed for TF binding sites. Such sites were predicted by MatInspector, a Genomatix program (http://www.genomatix.de) using TRANSFAC matrices (vertebrate matrix; core similarity 1.0 and matrix similarity 0.8) PROMO, which uses version 8.3 TRANSFAC (http://alggen.lsi.upc.es/cgi-bin/promo_v3/promo/promoinit.cgi?dirDB=TF_8.3) and TFBIND, which uses weight matrix in the database TRANSFAC R.3.4 (http://tfbind.hgc.jp).

### Small interfering RNA (siRNA) transfection in chicken PGCs

Chicken PGCs were seeded at a density of 2.5 × 10^5^ per well of a 12-well plate in 1 mL of medium. Then, the cells were transfected with each siRNA (50 pmol/L) using RNAiMAX (Invitrogen). Negative control siRNA with no complementary sequence in the chicken genome was used as a control. The sequence of each siRNA is listed in Table 2. After transfection for 48 h, total RNA was extracted using TRIzol reagent (Invitrogen). The knockdown efficiency of predicted TFs and their effects on the expression of germ cell-related genes including *CVH*, *cDAZL*, *CIWI*, and *CDH* were measured using quantitative reverse transcription-polymerase chain reaction (RT-PCR).

**Table 2:** List of small interfering RNA sequences used for knockdown analysis

	siRNA sequence (5′ → 3′)	
Target genes	Sense	Antisense
*EP300*-#1	GAGUUCUCCUCACUACGAA	UUCGUAGUGAGGAGAACUC
*EP300*-#2	GAUGAAUGCUGGCAUGAAU	AUUCAUGCCAGCAUUCAUC
*GABPA*-#1	GAGCAAGGUAUGUGUCUGU	ACAGACACAUACCUUGCUC
*GABPA*-#2	CACAAGAAGUCAACCAUCA	UGAUGGUUGACUUCUUGUG
*HSF2*-#1	GUGUUGGAUGAACAGAGAU	AUCUCUGUUCAUCCAACAC
*HSF2*-#2	CAGAACUGAGAGCAAAACA	UGUUUUGCUCUCAGUUCUG
*NFYA*-#1	UCAGACAGCUUACAGACUA	UAGUCUGUAAGCUGUCUGA
*NFYA*-#2	CAGUACACAGCCAACAGUA	UACUGUUGGCUGUGUACUG
*SP3*-#1	CAGUACAGUGCUGGCAUCA	UGAUGCCAGCACUGUACUG
*SP3*-#2	CUGGUAAUAUAGUACAGA	AUCUGUACUAUAUUACCAG
*ZNF143*-#1	GCAGCGUUUCAUAGCACCU	AGGUGCUAUGAAACGCUGC
*ZNF143*-#2	GAGCUUGAAAACUCCUAGU	ACUAGGAGUUUUCAAGCUC

### RNA isolation and quantitative RT-PCR

Total RNA of siRNA-treated PGCs was extracted using TRIzol reagent (Life Technologies, Carlsbad, CA, USA), in accordance with the manufacturer’s protocol. One microgram of each RNA was reverse-transcribed with the Superscript III First-strand Synthesis System (Invitrogen). The cDNA was diluted fourfold and used as a template for quantitative real-time PCR, which was performed using the Step One Plus real-time PCR system (Applied Biosystems) with EvaGreen (Biotium, Hayward, CA, USA). Each test sample was investigated in triplicate. Then, the relative gene expression of individual samples was calculated after normalization with glyceraldehyde 3-phosphate dehydrogenase (*GAPDH*) expression as an endogenous control [14, 27]. The primer pairs, which were designed using NCBI Primer-BLAST (https://www.ncbi.nlm.nih.gov/tools/primer-blast/index.cgi?LINK_LO C = BlastHome), used for the detection of cDNAs are listed in Table 3.

**Table 3:** List of primer sequences used for quantitative reverse transcription polymerase chain reaction

				Primer sequence (5′ → 3′)		
No.	Gene Symbol	Description	Accession No.	Forward	Reverse	Product Size, bp
1	*EP300*	E1A binding protein p300	XM_004937710.1	AGCTGCAGATGGAGGAGAAATC	ACGGTAAAGTGCCTCCAGTG	242
2	*GABPA*	GA binding protein transcription factor, alpha subunit 60 kDa	NM_001007858.1	TGAACAGGTGACACGATGGG	GGGACTCGCTGGAAGAAGTC	225
3	*HSF2*	heat shock transcription factor 2	NM_001167764.1	CCAGTTATCACCTGGAGCC	CCAACAAGTCCTCTCGACCC	242
4	*NFYA*	nuclear transcription factor Y, alpha	NM_001006325.1	TCAGCCACCTGTGAAGACAC	TCGAACTGGGCTTTCACCTC	231
5	*SP3*	Sp3 transcription factor	NM_204603.1	GGCAAAAGGTTCACTCGCAG	GTGTTGTTCCTCCCGCAGTA	229
6	*ZNF143*	zinc finger protein 143	XM_004941377.1	GAAGCGGCACATCCTTACCT	CCCTGACTTCCACAGCGATT	216

### Statistical analysis

All data are expressed as means ± standard deviation from three independent experiments. One-way analysis of variance with Bonferroni correction was used to calculate the significance of differences between experimental groups. GraphPad Prism software (ver. 5.0; GraphPad Software, La Jolla, CA, USA) was used to evaluate the data. *P < 0.05*; was considered statistically significant.

## Results

### Identification of the minimal promoter region for transcription of the *CVH* gene

**Fig. 1:**
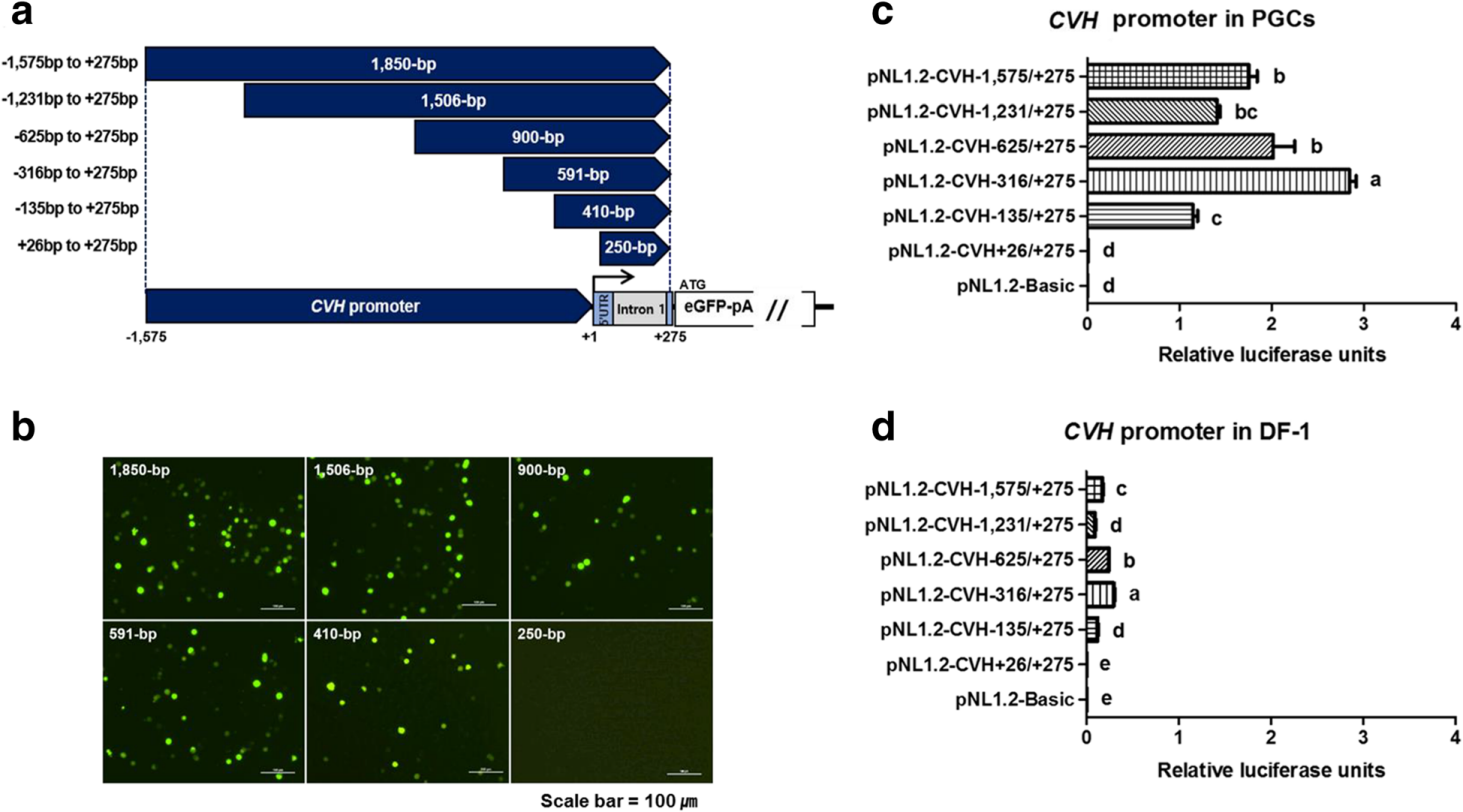
Identification of promoter region for inducing germ cell-specific gene expression in the chicken *vasa* homologue (*CVH*) promoter through 5′ deletion assays. **a** Schematic diagram of the constructed enhanced green fluorescent protein (eGFP) expression vectors with *CVH* promoters of different sizes. By 5′ deletion assays, six constructs including differently sized 5′ flanking sequences containing the 5′ untranslated region (UTR) were randomly designed. eGFP expression vector of a 250-bp fragment of the *CVH* promoter containing only the 5′ UTR. **b** Twenty-four hours after transfection, the expression of eGFP under the control of the differently sized promoters in cultured chicken primordial germ cells (PGCs) was monitored by microscopy. Each fragment used for driving eGFP expression was ligated into the NanoLuc luciferase expression vector (pNL1.2-Basic) to measure promoter activity. Dual luciferase assay of *CVH* promoter activity in PGCs (**c**) and DF-1 (**d**). NanoLuc luciferase expression levels were normalized to the luciferase activity of internal firefly control and are expressed as relative luciferase units. Scale bar = 100 μm. Different letters (a–e) indicate significant differences (*P* < 0.05)

To investigate the gene promoter for the expression of *CVH* mRNA, we constructed eGFP expression vectors containing different sizes of the *CVH* promoter by 5′ deletion, spanning a 1,850-bp region from the 5′ flanking region to the 5′ untranslated region (UTR) (Fig. 1a). Subsequently, we tested whether the differently sized *CVH* promoters can induce the expression of eGFP in cultured chicken PGCs. As shown in Fig. 1b, the following eGFP reporters were associated with the expression of green fluorescence in chicken PGCs: 1,850-bp fragment (−1,575/+275), 1,506-bp fragment (−1,231/+275), 900-bp fragment (−625/+275), 591-bp fragment (−316/+275), and 410-bp fragment (−135/+275); however, the smallest fragment (+26/+275) did not induce this expression. To evaluate the promoter activity further, we performed the dual luciferase reporter assay using the same fragments of the *CVH* promoter in chicken PGCs and DF-1. Consistent with the findings for eGFP expression, the luciferase reporters containing the promoter region of *CVH* presented strong enzyme activity, but we could not detect enzyme activity from the smallest 250-bp fragment and pNL1.2-basic, an empty vector (Fig. 1c). Notably, compared with DF-1 fibroblast cells, chicken PGCs generally presented at least 10 times higher luciferase activities (Fig. 1c and d). Collectively, these results suggest that the minimal promoter region of the *CVH* gene is located at −135 to +275 bp, which includes the 5′ UTR, and plays an important role in the transcription of this gene in chicken PGCs.

### Investigation of the *cis*-regulatory elements of the *CVH* gene

**Fig. 2:**
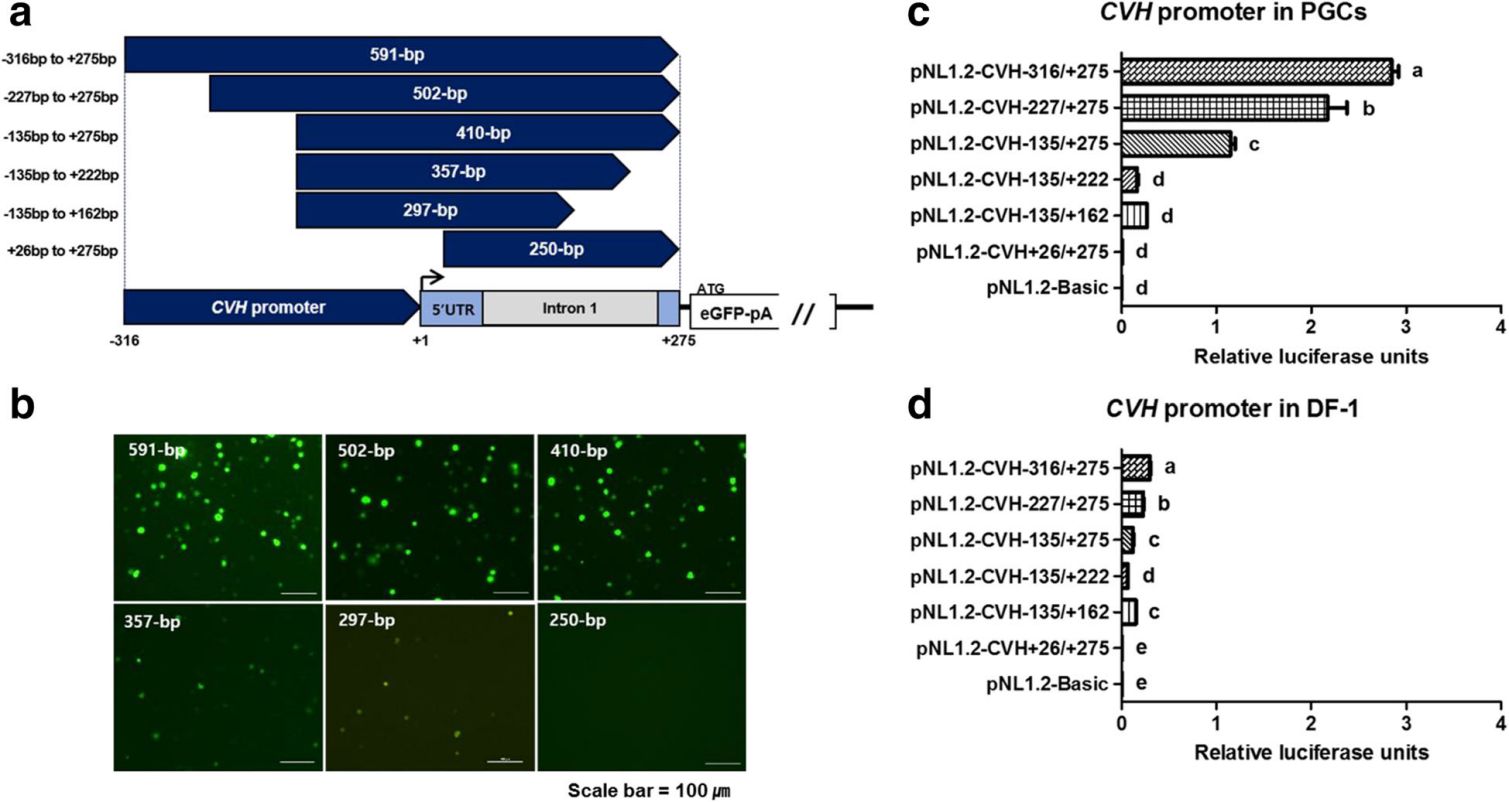
Identification of *CVH* promoter regions through 5′ and 3′ fragmentation assays. **a** Schematic diagram of six fragmented constructs of the *CVH* promoter. Constructs were designed for expression analysis using an eGFP expression vector: 5′ and 3′ random deletion assays were conducted for a 591-bp fragment (−316/+275) and a 410-bp fragment (−135/+275), respectively. For 3′ deletion for a 410-bp fragment (−135/+275), two constructs for a 357-bp fragment (−135/+222) and a 297-bp fragment (−135/+162) contained part of the intron region. **b** Expression analysis of eGFP under different fragments of the *CVH* promoter in cultured chicken PGCs. Twenty-four hours after transfection, the expression of eGFP was observed by fluorescence microscopy. Each fragment used for driving eGFP expression was ligated into the NanoLuc luciferase expression vector (pNL1.2-Basic) for measuring promoter activity. The expression of NanoLuc luciferase was measured using Nano-Glo-dual luciferase assays in PGCs (**c**) and DF-1 (**d**). Promoter activity was measured as the ratio of NanoLuc luciferase expression levels to internal firefly control and is expressed as relative luciferase units. Scale bar = 100 μm. Different letters (a–e) indicate significant differences (*P* < 0.05)

For further investigation of the potential transcriptional *cis-*elements in the *CVH* promoter, we performed 5′ and 3′ fragmentation assays using the 591-bp fragment (−316/+275) that presented the highest luciferase reporter activity, as well as a 410-bp fragment (−135/+275) (Fig. 2a). First, we confirmed the eGFP expression with the designed fragments of the *CVH* promoter in chicken PGCs. Among six fragment constructs, the 591-bp fragment (−316 /+275), 502-bp fragment (−227/+275), and 410-bp fragment (−135/+275) were associated with the strong expression of green fluorescence in chicken PGCs compared with the 357-bp fragment (−135/+222) and the 297-bp fragment (−135/+162). These latter two fragments (357-bp and 297-bp fragments) still showed minimal promoter activity, while the 250-bp fragment (+26/+275) showed none (Fig. 2b). We also conducted a dual luciferase reporter assay using NanoLuc luciferase expression vectors to compare the *CVH* promoter activity in chicken PGCs and DF-1. As shown in Fig. 2c, deletion of the 92-bp fragment between −227/+275 bp and −135/+275 bp resulted in a dramatic decrease in luciferase activity. These results suggest that a positive transcriptional *cis*-element is located in this region. Furthermore, partial deletion of the 5′ UTR including intron 1 (−135/+222 bp and −135/+162 bp) also produced a dramatic change in promoter activity (Fig. 2c). Interestingly, all tested fragments showed higher luciferase activity in PGCs than in DF-1 fibroblasts (Fig. 2d). Collectively, these results indicate that the PGC-specific gene expression requires at least a 410-bp sequence of the 5′ upstream region of the *CVH* gene along with the 5′ UTR including intron 1.

### Prediction and selection of the TFs involved in transcriptional control of the *CVH* promoter in chicken PGCs

**Fig. 3:**
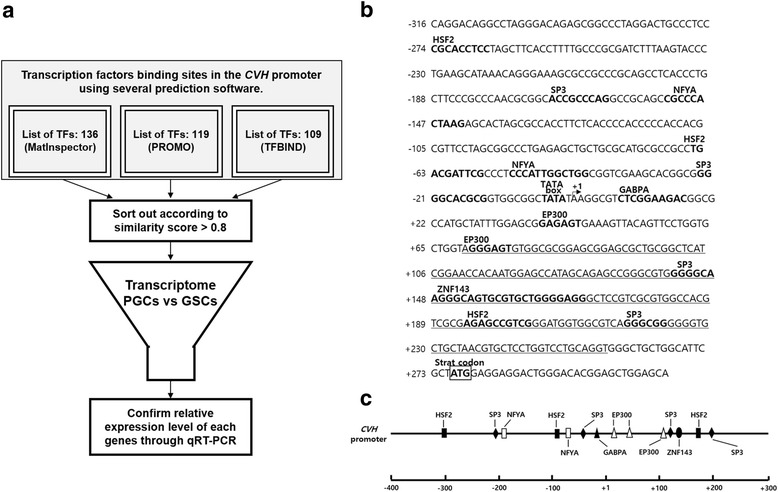
Location of predicted transcription factor (TF) binding sites on the *CVH* promoter. **a** A flowchart of the process of selection of TFs having putative binding sites in the 591-bp fragment (−316/+275) of the *CVH* promoter. The input is sets of TFs predicted by several prediction software programs (MatInspector, PROMO, and TFBIND). TFs with a similarity score below 0.8 were then removed. The putative TFs were extracted depending on significant expression in chicken PGCs, from the transcriptome data. **b** Nucleotide sequences of the 5′ flanking region of the 591-bp fragment (−316/+275) of the *CVH* promoter. Selected TFs were marked on the sequences of the *CVH* promoter region. **c** Schematic diagrams of the locations of these predicted factors in the promoter region. +1 indicates the transcriptional initiation site and boxed ATG indicates the translational start site. The bold sequences show predicted binding sites of the TFs. Underline indicates the intron region of the *CVH* gene. GSCs, gonadal stromal cells

Based on the findings of the *CVH* promoter activity mentioned above, we predicted TFs that have binding sites in the 591-bp fragment (−316/+275) of the *CVH* promoter using three software programs (PROMO, TFBIND, and MatInspector). Additionally, we attempted to clarify the TFs that were more highly expressed in chicken PGCs than in other cell types, such as Stage X blastodermal cells, gonadal stromal cells (GSCs), and chicken embryonic fibroblasts (CEFs) using previously obtained transcriptome data (Fig. 3a) [3, 4, 13]. From these analyses, we identified six TFs (*EP300*, *GABPA*, *HSF2*, *NFYA*, *SP3*, and *ZNF143*) that were expressed at significantly higher levels in PGCs and have putative binding sites in the 591-bp fragment (−316/+275) of the *CVH* promoter. To summarize our findings, we marked the consensus sequences and positions of the predicted TFs in sequences of the *CVH* promoter including TATA-box sequence and transcription start codon in Fig. 3b and c.

### Predicted TFs affecting the transcriptional activity of germ cell-specific RBPs

**Fig. 4:**
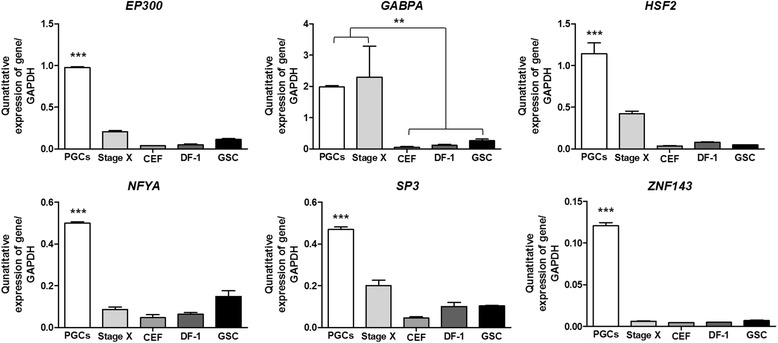
Quantitative expression analysis of predicted TFs in various cell types. By quantitative reverse transcription-polymerase chain reaction (qRT-PCR) analysis, predicted TFs were analyzed with the prepared PGCs, Stage X blastoderm, chicken embryonic fibroblasts, DF-1, and GSCs. Error bars indicate the standard deviation of triplicate analysis. Significant differences are indicated as ***P < 0.001 and **P < 0.01

**Fig. 5:**
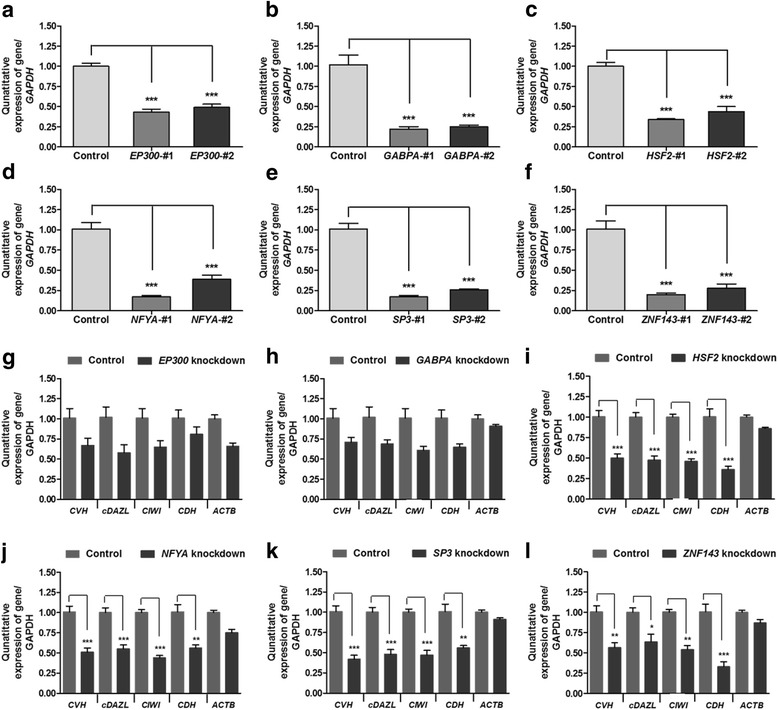
Relative gene expression analysis after knockdown of predicted TFs in cultured PGCs. **a**–**f** Confirmation of siRNA knockdown efficiency in vitro in chicken PGCs for the predicted TFs as determined by qRT-PCR. **g**–**l** Relative expression analysis of germ cell-related RNA binding proteins (*CVH*, c*DAZL*, *CIWI*, and *CDH*) in cultured PGCs after treatment of each siRNA. *ACTB* (*beta-actin*) was used as a control for silencing the specificity of the knockdown probes. qPCR was conducted in triplicate, with data being normalized to the expression of *GAPDH* as a control. Significant differences between control and treatment groups are indicated as ***P < 0.001, **P < 0.01, and *P < 0.05. Error bars indicate the standard error of triplicate analyses

To confirm the expression of the selected TFs in chicken PGCs, we conducted quantitative RT-PCR using the RNA samples prepared from various cells/tissues (PGCs, Stage X, CEFs, DF-1, and GSCs). The results showed that the expression of five TFs is highly PGC-specific, with the exception being *GABPA*, which is expressed in both PGCs and Stage X equally (Fig. 4). These results indicate that these TFs may be involved in transcriptional control of the *CVH* promoter by directly interacting with it in chicken PGCs. We further examined whether these TFs affect the transcription of germ cell-specific RBPs (*CVH*, *cDAZL*, *CIWI*, and *CDH*) in chicken PGCs using a siRNA-mediated knockdown assay. As shown in Fig. 5, in the samples with the highest knockdown efficiency of *HSF2*, *NFYA*, *SP3*, and *ZNF143* mRNA expression in chicken PGCs, the expression of RBP mRNA was significantly reduced, suggesting that these TFs function in regulating transcriptional control of the *CVH* promoter, and other PGC-specific RBPs, such as *cDAZL*, *CIWI*, and *CDH*, while *EP300* and *GABPA* remain unaffected. Taken together, these results suggest that these TFs (*HSF2*, *NFYA*, *SP3*, and *ZNF143*) play a role in the transcription of PGC-specific RBPs through direct binding to 5′ upstream promoter regions.

## Discussion

The results of the current study suggest that the promoter region of the *CVH* gene, which extends from −316 to +275 bp and contains the 5′ UTR and intron 1, can control the transcription of the *CVH* gene in chicken PGCs. They also suggest that significantly upregulated TFs such as *HSF2*, *NFYA*, *SP3*, and *ZNF143* in chicken PGCs play a role in expression of the *CVH* gene by directly interacting with putative binding sites of the *CVH* gene promoter.

VASA, an evolutionarily conserved RBP that promotes translational control of germ cell-specific genes, is expressed specifically in germ cells during germline development [28]. Several reports have shown that *vasa* play a critical role in the formation of the germplasm and gametogenesis in invertebrates such as *Caenorhabditis elegans* and *Drosophila melanogaster* [29, 30]. In addition, VASA expression in germ cells is essential for their survival and proliferation [31, 32]. In transgenic animals, 5.1-kb, 4.7-kb, 2.4-kb, 5.6-kb, 8-kb and 4.3-kb of *vasa* promoter have been used for germ cell specific expression of reporter genes in medaka [33], rainbow trout [34], zebrafish [35], mice [36], cows [37] and pig [38], respectively. Moreover, in *Drosophila melanogaster*, it is reported that germline specific *vas* gene expression in oogenesis is required for a 40-bp genomic region of the *vas* gene though interacting specifically with certain ovarian protein [39]. In addition, in the malaria mosquito, *vasa*-like gene is specifically expressed in both the male and female gonads in adult mosquitoes and is characterized the regulatory regions that are the entire 5’UTR and only 380-bp of upstream sequence for the specific germline expression in the GSCs of both sexes [40]. Although studies on the transcriptional control of *CVH* for temporal and spatial regulation hold great promise for practical applications, regarding using a germ cell-specific promoter for tracing germ cells as well as understanding the molecular network of transcriptional regulation behind their unique characteristics, very limited information is available on the regulatory elements involved in transcriptional control of the *CVH* gene in chicken.

Previous study showed that the *CVH* gene requires a 5′ flanking region of 1,555-bp for higher induction of specific expression in germ cells at the transcriptional level [16]. However, as shown in Fig. 1, we described that the highest promoter activity region of the *CVH* gene, which is a 591-bp fragment (−316/+275) containing the 5′ UTR, is sufficient for the induction of specific expression in chicken PGCs, as determined by a 5′ deletion assay. Additionally, our findings demonstrate for the first time that the 5′ UTR containing intron 1 is required for promoter activity of the *CVH* gene in chicken PGCs, as determined through 5′ and 3′ fragmentation assays (Fig. 2). With regard to the roles of introns in transcriptional control in diverse organisms, several reports have shown that introns play a pivotal role in controlling transcription, including that of germline-specific genes, and act as enhancers to control gene expression [41–47]. In search for regulatory elements of *vasa* promoter in medaka, Li et al. demonstrated that the first intron plays an important role in the VAS activity from total of 11 regions identified within the 5.1-kb *vasa* promoter [47], and subsequently found that the first 35-bp of exon 1 of *vasa* gene is sufficient to increase transcriptional activity as a enhancer [48]. Therefore, it seems likely that the 5′ UTR containing intron 1 of the *CVH* gene would be valuable for constructing a germ cell-specific *CVH* promoter vector for the practical utilization of genetic resources.

Transcriptional control is required for regulatory elements such as specialized promoter sequences and promoter recognition *trans*-acting factors [49]. But, there are few reports on transcriptional regulators of *vasa* gene. A previous study revealed that Mitf acts as transcriptional activator of germ cell-specific genes encoded RNA-binding proteins such as *vasa*, *dazl* and *dnd* in medaka spermatogonial cell line [50]. Therefore, we investigated whether predicted TFs that have putative binding sites in the 591-bp fragment (−316/+275) of the *CVH* promoter can directly regulate the expression of chicken *CVH*. Using our previous transcriptome analysis, we identified TFs that were more highly expressed in chicken PGCs than in other cell types. The integrated approaches used in this study were complementary for finding novel TFs with putative binding sites in the *CVH* promoter. However, we could not find Mitf in the analyzed *CVH* promoter above mentioned. Finally, we selected six TFs (*EP300*, *GABPA*, *HSF2*, *NFYA*, *SP3*, and *ZNF143*) that have putative binding sites in the 591-bp fragment (−316/+275) of the *CVH* promoter through a series of experiments. In Fig. 3, all TFs are marked for consensus sequences and positions in the sequence of the *CVH* promoter, including the TATA-box sequence for transcriptional initiation and the start codon. We also validated their expression levels using quantitative RT-PCR. Based on the results, five TFs (*EP300*, *HSF2*, *NFYA*, *SP3*, and *ZNF143*) were significantly expressed in PGCs compared with their levels in other samples, while *GABPA* were significantly expressed in PGCs compared with CEF, DF-1, and GSC, but showed no significant difference in expression compared with that at Stage X (Fig. 4). We further examined whether these TFs affect the transcription of germ cell-specific RBPs (*CVH*, *cDAZL*, *CIWI*, and *CDH*) in chicken PGCs through siRNA-mediated knockdown.

With regard to significant expression and functions in germ cells, it has been reported that heat shock factor 2 (HSF2) plays a role during embryonic development and under stress conditions, prevents the formation of damaged gametes, and ensures the integrity of the reproductive process [51]. In addition, knockout mouse models have shown that HSF2 is involved in oogenesis and spermatogenesis and strongly expressed in PGCs [52, 53]. As a general transcription activator, NF-Y binds strongly at CCAAT motifs and consists of NF-YA, −YB, and -YC subunits [54]. In *C. elegans*, mutations in *nfya-1* affect the development of germ cells and also reduce the number of sperm [55]. Moreover, NF-Y is greatly affected by the CCAAT motif in terms of its transcriptional activity regarding *Miwi* and *CIWI* gene [56, 57]. Additionally, SP3 is one of the Sp family of TFs, which is characterized by three conserved zinc fingers [58], and positively or negatively controls the transcriptional activity of numerous genes through binding to the GC box in *cis*-regulatory elements [59]. Importantly, it has been reported that regulation of *Nanog* gene expression is required for Sp1 and Sp3 expression, besides Oct4 and Sox2, in mouse [60]. Zinc finger protein 143 (ZNF143) was first identified in *Xenopus* [61], and most ZNF143 binding sites are disturbed in promoters associated with CpG islands near the transcription start site in the mammalian genome [62]. *Znf143* is particularly expressed in the mouse ICM [63] and its expression has been implicated in the regulation of mammalian embryonic stem cell survival and renewal [64]. It was also proven that ZNF143, interacting with Oct4, governs Nanog expression through direct binding to the *Nanog* proximal promoter [65]. In addition, ZNF143 has recently been identified as a new factor connecting promoters and distal regulatory elements as an insulator function for lineage-specific gene expression [66]. With regard to transcriptional regulator of Mouse Vasa Homologue (MVH), *Znf143* preferred histone H3 lysine 27 acetylation (H3K27ac)-marked regions associated with the early genes including *Kit*, *Prdm1*, and *Sox2* rather than the late genes such as *Mvh*, *Piwil1*, *Piwil2*, *Tdrd7*, and *Tdrd9* in germ cells [67]. However, our results revealed that *HSF2*, *NFYA*, *SP3*, and *ZNF143* would be expected to function as transcriptional regulators in chicken PGCs. Collectively, our results demonstrate for the first time that these TFs are involved in promoter activity of germ cell-specific RBPs in chicken PGCs; however, it remains to be determined whether these TFs directly act on each gene promoter during chicken PGC development.

## Conclusion

In conclusion, we have identified the promoter region of the *CVH* gene for PGC-specific gene expression and found TFs such as *HSF2*, *NFYA*, *SP3*, and *ZNF143* associated with transcriptional control of the *CVH* gene in chicken PGCs. This information should aid a wide range of studies in constructing germ cell-specific synthetic promoters for tracing germ cells using transgenesis, as well as our understanding of the transcriptional regulation that maintains germness in PGCs.

## Abbreviations

ACTB: Beta-actin
bFGF: Basic fibroblast growth factor
cDAZL: Chicken DAZL
CDH: Chicken dead end homologue
CEF: Chicken embryonic fibroblast
CIWI: Chicken PIWI-like protein 1
CVH: Chicken vasa homolog
DAZL: Deleted in azoospermia-like
DMEM: Dulbecco’s modified eagle medium
eGFP: Enhanced green fluorescent protein
EP300: E1A binding protein p300
FBS: Fetal bovine serum
GABPA: GA binding protein transcription factor, alpha
GADPH: Glyceraldehyde 3-phosphate dehydrogenase
GSC: Gonadal stromal cell
H3K27ac: Histone H3 lysine 27 acetylation
HSF2: Heat shock transcription factor 2
ICM: Inner cell mass
NFYA: Nuclear transcription factor Y, alpha
PGC: Primordial germ cell
piRNAs: PIWI-interacting RNAs
PIWI: P-element induced wimpy testis
qRT-PCR: Quantitative reverse transcription-polymerase chain reaction
RBP: RNA binding protein
siRNA: Small interfering RNA
SP3: Stimulating protein 3
TF: Transcription factor
UTR: Untranslated region
ZNF143: Zinc finger protein 143
